# Hospital acquired pressure injury prediction in surgical critical care patients

**DOI:** 10.1186/s12911-020-01371-z

**Published:** 2021-01-06

**Authors:** Jenny Alderden, Kathryn P. Drake, Andrew Wilson, Jonathan Dimas, Mollie R. Cummins, Tracey L. Yap

**Affiliations:** 1 University of Utah College of Nursing, 10 S 2000 E, Salt Lake City, UT 84112 USA; 2 Department of Computer Science, Boise State University, 777 W Main Street, Boise, ID 83704 USA; 3 Parexel, 2520 Meridian Parkway, Durham, NC USA; 4 Duke University School of Nursing, 307 Trent Drive, Durham, NC 27710 USA

**Keywords:** Pressure ulcer/pressure injury, Risk assessment, Hospital-acquired condition

## Abstract

**Background:**

Hospital-acquired pressure injuries (HAPrIs) are areas of damage to the skin occurring among 5–10% of surgical intensive care unit (ICU) patients. HAPrIs are mostly preventable; however, prevention may require measures not feasible for every patient because of the cost or intensity of nursing care. Therefore, recommended standards of practice include HAPrI risk assessment at routine intervals. However, no HAPrI risk-prediction tools demonstrate adequate predictive validity in the ICU population. The purpose of the current study was to develop and compare models predicting HAPrIs among surgical ICU patients using electronic health record (EHR) data.

**Methods:**

In this retrospective cohort study, we obtained data for patients admitted to the surgical ICU or cardiovascular surgical ICU between 2014 and 2018 via query of our institution's EHR. We developed predictive models utilizing three sets of variables: (1) variables obtained during routine care + the Braden Scale (a pressure-injury risk-assessment scale); (2) routine care only; and (3) a parsimonious set of five routine-care variables chosen based on availability from an EHR and data warehouse perspective. Aiming to select the best model for predicting HAPrIs, we split each data set into standard 80:20 train:test sets and applied five classification algorithms. We performed this process on each of the three data sets, evaluating model performance based on continuous performance on the receiver operating characteristic curve and the F_1_ score.

**Results:**

Among 5,101 patients included in analysis, 333 (6.5%) developed a HAPrI. F_1_ scores of the five classification algorithms proved to be a valuable evaluation metric for model performance considering the class imbalance. Models developed with the parsimonious data set had comparable F_1_ scores to those developed with the larger set of predictor variables.

**Conclusions:**

Results from this study show the feasibility of using EHR data for accurately predicting HAPrIs and that good performance can be found with a small group of easily accessible predictor variables. Future study is needed to test the models in an external sample.

## Background

Hospital-acquired pressure injuries (HAPrIs), formerly called pressure ulcers or bedsores, are areas of injury to the skin and underlying tissue caused by external pressure, usually over a bony area. In the United States, costs attributable to HAPrIs exceed $26 billion a year [1]. HAPrIs are considered mostly preventable and defined as a "never event" or "serious reportable event" by the National Quality Forum [2]. HAPrIs occur among 5–10% of critical-care patients [3], with the highest risk among surgical critical-care patients [4].

Most HAPrIs are preventable; however, prevention may require using measures not feasible for every patient because of cost or nursing time [5]. Therefore, recommended standards of practice include pressure injury (PrI) risk assessment at each nursing shift and with changes in patient status [6]. Unfortunately, the risk-assessment tools currently in use, such as the widely used Braden Scale [7], classify most critical-care patients as "high risk" [8–13], and therefore do not give critical-care nurses the information they need to allocate limited PrI prevention resources appropriately. A PrI risk-assessment tool allowing nurses to differentiate PrI risk among critical-care patients is imperative.

Electronic health records (EHRs) and data analytics can improve HAPrI risk assessment. Recent advances in machine learning (ML) present an opportunity to modernize and enhance future HAPrI risk assessment using the extensive data readily available in the EHR. Risk assessment and predicting future events are areas where combining modern ML techniques may identify novel patterns unapparent to humans to predict a target (in our case, HAPrI development). The benefits of ML approaches are particularly relevant in the ICU setting because of the dynamic physiologic nature of critical care patients. Unlike traditional prognostic tools like the Braden Scale, an ML approach can incorporate non-linear, complex interactions among variables (including correlated variables) [14].

The purpose of the current study was to develop and compare models predicting HAPrIs among surgical critical-care patients using EHR data. The specific aims were to (1) develop and compare predictive models, and (2) develop and compare more parsimonious predictive models using data readily available—and easily accessible—in the EHR.

## Methods

### Design

We chose a retrospective cohort design. All data were entered into structured fields in the EHR (EPIC^©^) and then obtained via a query from our institution's enterprise data warehouse (EDW).

### Sample

Adult patients (aged ≥ 18 years) who were admitted to the surgical intensive care unit (SICU) or cardiovascular surgical intensive care unit (CVICU) at a Level 1 trauma center and academic medical center in the western United States between 2014 and 2018 were included in the sample. We included patients with community-acquired (present on admission) PrIs because those patients have risk for subsequent HAPrIs [15]. We excluded patients admitted to the ICU for less than 24 h because these short-stay patients were unlikely to manifest a HAPrI with this duration of exposure [16]. In those patients with multiple ICU stays, our data were limited to the first ICU stay. At our facility, all ICU patients are placed on pressure redistribution mattresses [17].

### Measures

#### HAPrI outcomes

There are six stages of HAPrIs defined by the National Pressure Injury Advisory Panel (NPIAP) [18]. Stage 1 HAPrIs are areas of nonblanching redness or discoloration in intact skin. Stage 2 HAPrIs represent partial-thickness tissue loss with exposed, viable dermis. Stage 3 HAPrIs are full-thickness wounds not extending into muscle, bone, or tendon. Stage 4 HAPrIs are full-thickness wounds extending down to muscle, tendon, or bone. Deep-tissue injuries (DTIs) are areas of intact or nonintact skin with a localized area of persistent, nonblanchable, deep-red, maroon, or purple discoloration revealing a dark wound bed or blood-filled blister. Finally, unstageable HAPrIs are areas of full-thickness tissue loss. These cannot be evaluated because eschar or slough obscures the area. Among these stages, only Stage 1 HAPrIs are reversible, sometimes resolving within hours after pressure offloading.

The outcome variable was the development of a HAPrI (Stages 2–4, DTI, or unstageable injury); Stage 1 PrIs were not included because they are often reversible, sometimes healing within hours [19, 20]. We used the initial (first recorded) HAPrI for patients with multiple injuries. Nurses at our hospital conduct a head-to-toe skin inspection once per shift, recording information about HAPrIs in a structured field in the EHR (EPIC^©^). We used the structured fields from the nursing documentation section of the EHR to record HAPrIs. We chose to use the structured fields from nursing documentation because a prior study showed that International Classification of Diseases codes underreport HAPrIs [21], likely because HAPrIs are noticed by nurses during head-to-toe skin assessments but not always flagged for inclusion in physician notes or billing codes. Furthermore, we relied on nursing documentation because it is the only accurate source of data for the date and time the HAPrI developed. All possible HAPrIs recorded in the nursing documentation at our facility are verified by a certified wound nurse, thus ensuring accuracy.

#### Potential predictor variables

Using Coleman and colleagues' conceptual framework for PrI etiology [22], we conducted a systematic review of the literature aimed at identifying risk factors for HAPrIs among critical-care patients [3]. Additionally, we conducted interviews with subject-matter experts, including clinicians at our study site: wound nurses, ICU nurses, intensivist physicians, surgeons, dieticians, physical therapists, nursing assistants, anesthesiologists, and other healthcare team members. The result of this formative work is reflected in Table 1.

**Table 1:** Predictor variables

Laboratory data		
L_1_	Serum lactate	Maximum serum lactate (mg/dL)
L_2_	Serum creatinine	Maximum serum creatinine (mg/dL)
L_3_	Serum glucose	Maximum serum glucose (mg/dL)
L_4_	Hemoglobin	Minimum hemoglobin (mg/dL)
L_5_	Serum albumin	Minimum serum albumin (mg/dL)
L_6_	Arterial PaO2	Minimum arterial partial pressure of oxygen (mmHg)
L_7_	Arterial pH	Minimum arterial (pH)
Nursing skin assessment data		
N_1_	Thin epidermis	Thin epidermis with subcutaneous tissue loss
N_2_	Skin tear	Presence of a skin tear
N_3_	Community acquired pressure injury	Community-acquired pressure injury present at admission
Surgical time		
S_1_	Longest surgery	Longest single surgery, measured from start of anesthesia to stop of anesthesia
Vasopressor infusions		
V_1_	Vasopressin dose	Highest dose of vasopressin (units/min)
V_2_	Norepinephrine dose	Highest dose of norepinephrine infusion (mcg/kg/min)
V_3_	Norepinephrine infusion	Norepinephrine received (yes/no)
V_4_	Epinephrine infusion	Epinephrine received (yes/no)
V_5_	Phenylephrine infusion	Phenylephrine received (yes/no)
V_6_	Dopamine infusion	Dopamine received (yes/no)
V_7_	Vasopressin infusion	Vasopressin received (yes/no)
Other potential predictors		
O_1_	MEWS score	Maximum Modified Early Warning score
O_2_	GCS	Minimum Glasglow Coma Scale score
O_3_	Fluid status	Maximum daily net intake (in L)
O_4_	Length of ICU stay prior to HAPrI	Length of stay in the ICU prior to HAPrI development
O_5_	Riker score	Minimum Riker Sedation and Agitation Scale Score
O_6_	BMI	Admission Body Mass Index (kg/m^2^)
Braden Scale Scores		
B_1_	Braden Scale total	Minimum total Braden Scale Score
B_2_	Braden Mobility Subscale	Minimum Braden Mobility Subscale Score
B_3_	Braden Friction and Shear	Minimum Braden Friction and Shear Subscale Score

**Fig. 1:**
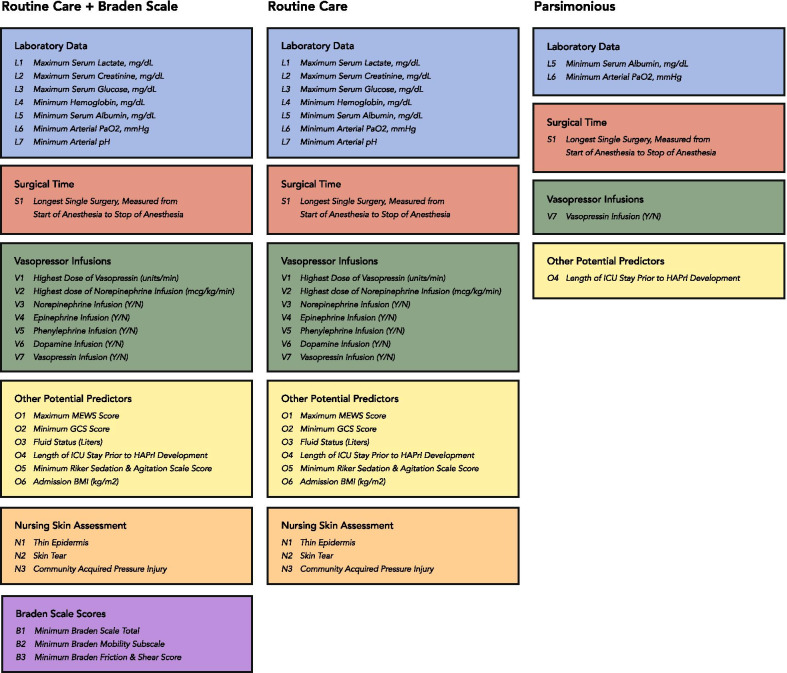
Datasets

We developed our predictive models utilizing three different sets of variables, depicted in Fig. 1. The first set included all of the available data: data produced in the EHR during routine care and Braden Scale scores recorded by nurses. The second set of predictor variables included only data produced during routine care (excluding Braden Scale scores). The third data set had a parsimonious group of five variables we selected because they were easily accessible in EHRs from a data-warehouse perspective.

### Analysis

#### Data preprocessing

We obtained data from our study site's EDW; the query was performed by a team of biomedical informaticists. We limited our data to events occurring before the HAPrI outcome to mitigate target leakage (also called data leakage). Target leakage happens when a predictive model includes information unavailable when the model is applied to prospective data.

By validating the data in the human-readable system, we ensured that the query was accurate in terms of variable definitions and meaning and date/time stamps. A practicing critical-care nurse who worked within the EPIC^©^ EHR system compared information obtained in the query against data in the human-readable system for 30 randomly selected participants, including 15 participants with HAPrIs. We chose the number 30 because, in earlier work conducted within a legacy EHR system, it was necessary to review up to 10 cases to find some inconsistencies; we tripled that figure to be conservative [23].

#### Missing data

Missing data were quantified and then assessed for patterns of missingness using graphical clustering displays. For variables not informatively missing, we replaced the missing data with values found using single-value random forest imputation. For clinically informatively missing variables, known by clinicians to be associated with increased severity of illness, we substituted a "0" for missing and a "1" for not missing.

#### Imbalanced classes

Due to the nature of the data, there is a strong class imbalance of HAPrIs; more specifically, the majority of patients in ICUs do not develop HAPrIs, and this can present a problem when applying ML methods for prediction. For example, in models designed to optimize accuracy, the resulting predictive algorithms can end up classifying all outcomes as belonging to the majority class, and the accuracy of the model will be considered high. To remedy the high-class imbalance, we employed synthetic minority oversampling technique (SMOTE) in the training data [24]. By applying SMOTE to the data before the algorithms, we ensured that models would better classify the outcomes in the minority class.

In addition to sensitivity and specificity, we considered both precision (also called positive predictive value) and recall (also called sensitivity) in evaluating model performance. The precision and recall of a model focus on the proportion of true positives and are calculated by

1 $$precision = \frac{TPs}{{TPs + FPs}}$$

and

2 $$recall = \frac{TPs}{{TPs + FNs}}$$

In words, precision is the ratio of correctly predicted positives to the total predicted positives, whereas recall is the ratio of correctly predicted positives to the total actual positives. The F_1_ score is a summary score accounting for both precision and recall, defined as

3 $$F_{1} Score = 2 \cdot \frac{precision \cdot recall}{{precision + recall}}$$

This quantity is the harmonic mean of precision and recall and preferable over a simple average because it penalizes extreme values.

**Fig. 2:**
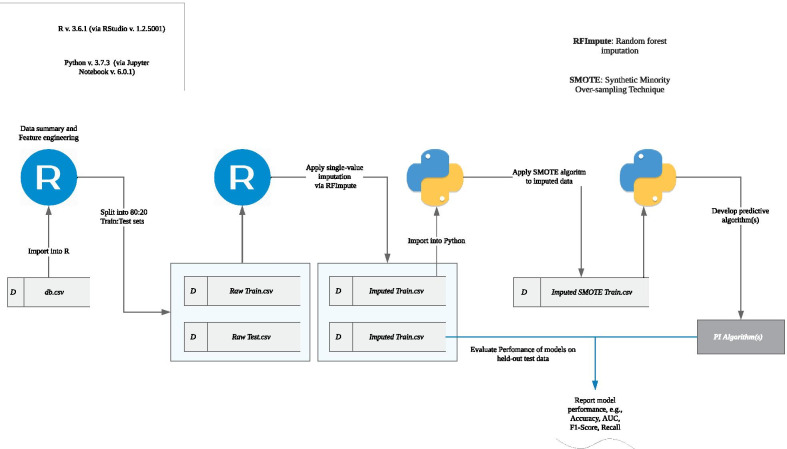
Data analysis workflow

All post-EPIC^©^ data preparation (frequencies, assessment of missingness, imputation) was performed in *R* version 3.6.1. The SMOTE procedure and model development and evaluation were performed using *Python* version 3.7.0. Figure 2 depicts the data-analysis workflow.

#### Statistical analysis

To identify factors associated with HAPrIs, we conducted univariate analysis. We used a Mann–Whitney U test for ordinal variables, a *t*-test or Mann–Whitney U test for parametric versus nonparametric continuous variables, and a Pearson chi-square or Fisher exact test, as appropriate, for categorical variables.

For any given prediction or estimation problem, it is not possible beforehand to know the true form of relationships between variables and, therefore, not possible to know a priori which learning algorithm would be best. This can be particularly true when relationships between variables are complex, non-linear, and nested within each other. For this reason, our approach was to apply different types of models to different sets of variables (features) with different pre-processing techniques and compare the models' performance. The performance of each model was evaluated both as a binary classifier using a confusion matrix and having continuous predicted probabilities for true positive and negative cases plotted as receiver operator characteristic (ROC) curves, with estimates of area under such curves (AUC). No post-prediction calibration was performed for generated models, although distributions of predicted probabilities were assessed (and probabilities from logistic regression are already naturally calibrated). Candidate models included keras (neural networks, with two hidden layers, each with 20 nodes each, and one output layer with a single node), random forests (ensembles of decision trees), gradient and adaptive (AdaBoost) boosting (algorithm focuses more on the examples that previous [internal] 'weak learners' misclassified, i.e., focuses more on *hard-to-train cases*), and multivariable logistic regression. All data processing and analysis steps were performed in Python v. 3.7 with all machine learning models available from the sklearn library [25]. For reproducibility and transparency, all code and results are hosted on and accessible through our Open Science Framework (OSF) page, https://tinyurl.com/OSF-Predicting-HAPrI.

## Results

### Sample characteristics

Our final sample consisted of 5,101 adult SICU and CVICU patients. One patient was excluded due to the absence of a medical record number; without the medical record number, we could not access data for other variables. The sample was predominantly male (*n* = 3302, 65%) and White (*n* = 4256, 83%). The mean age was 58 years (*SD* = 17).

### HAPrI outcome

HAPrIs developed among 333 patients (6.5%), which is consistent with our institution's quarterly prevalence surveys.

### Predictor variables

Table 2 outlines the univariate relationships between predictor variables and HAPrI outcomes. However, individual variable significance was not used to guide model development. Variables sets were selected based on ontologies presented in Fig. 1.

**Table 2:** Potential predictor variables and hospital-acquired pressure injury development

Variable	All patients	Patients with no HAPrI *n* = 4768	Patients with a HAPrI *n* = 333	*p* value
Demographic data				
Age in years, *M* (*SD*)	57.8 (17%)	58 (17%)	58 (15%)	*p* = .74
Sex, male, *n* (%)	3302 (65%)	3086 (65%)	216 (65%)	*p* = 1.0
Race, White, *n* (%)	4256 (83%)	3988 (84%)	268 (81%)	*p* = .13
Ethnicity, non-Hispanic, *n* (%)	4452 (87%)	4166 (87)	286 (86%)	*p* = .54
Laboratory data				
Serum lactate (mg/dL), *M* (*SD*)	4.0 (3.7)	3.9 (3.6)	5.9 (5.0)	*p* < .001
Serum creatinine (mg/dL), *M* (*SD*)	1.9 (1.9)	1.8 (1.9)	2.8 (2.1)	*p* < .001
Serum glucose (mg/dL), *M* (*SD*)	231 (148)	228 (143)	285 (200)	*p* < .001
Hemoglobin (mg/dL), *M* (*SD*)	9.0 (2.6)	9.1 (2.6)	7.6 (2.2)	*p* < .001
Serum albumin (mg/dL), *M* (*SD*)	3.1 (0.8)	3.2 (0.7)	2.7 (0.6)	*p* < .001
Arterial PaO2 (mmHg), *M* (*SD*)	54 (40)	55 (40)	46 (31)	*p* < .001
Arterial pH, *M* (*SD*)	7.27 (0.11)	7.27 (0.10)	7.22 (0.13)	*p* < .001
Nursing skin-assessment data				
Thin epidermis, *n* (%)	882 (18%)	801 (17%)	81 (24%)	*p* = .001
Skin tear, *n* (%)	641 (13%)	548 (11%)	93 (28%)	*p* < .001
Community-acquired pressure injury, *n* (%)	168 (3%)	138 (2.9%)	30 (9%)	*p* < .001
Surgical time				
Longest surgery, minutes, *M* (*SD*)	181 (156)	179 (152)	204 (197)	*p* < .001
Vasopressor infusions				
Vasopressin dose (units/min), *M* (*SD*)	0.01 (0.03)	0.01 (0.03)	0.02 (0.03)	*p* < .001
Norepinephrine dose (mcg/kg/min), *M* (*SD*)	0.06 (0.42)	0.05 (0.29)	0.25 (1.18)	*p* < .001
Norepinephrine (yes/no), *n* (%)	1154 (23%)	982 (21%)	172 (52%)	*p* < .001
Epinephrine (yes/no), *n* (%)	1490 (29%)	1352 (28%)	138 (41%)	*p* < .001
Phenylephrine (yes/no), *n* (%)	190 (4%)	173 (4%)	17 (5%)	*p* = .22
Dopamine (yes/no), *n* (%)	205 (4%)	186 (4%)	19 (5%)	*p* = .14
Vasopressin (yes/no), *n* (%)	1757 (34%)	1586 (33%)	171 (51%)	*p* < .001
Other potential predictors				
MEWS score, *M* (*SD*)	4.3 (1.9)	4.1 (2.0)	6.2 (1.9)	*p* < .001
GCS, *M* (*SD*)	9.2 (4.8)	9.3 (4.7)	6.8 (4.6)	*p* < .001
Fluid status (liters), *M* (*SD*)	2.3 (2.1)	2.2 (2.0)	3.7 (2.6)	*p* < .001
Length of ICU stay prior to HAPrI (days), *M* (*SD*)	5 (7)	4.6 (6.2)	13 (9.9)	*p* < .001
Riker score, *M* (*SD*)	2.8 (1.2)	2.9 (1.2)	2.1 (1.2)	*p* < .001
BMI (kg/m^2^), *M* (*SD*)	30.1 (12.4)	30.1 (12.5)	30.7 (11.0)	*p* = .36
Braden Scale scores				
Braden Scale total score, *M* (*SD*)	13 (3)	13 (3)	11 (3)	*p* < .001
Braden Mobility subscale, *M* (*SD*)	2.0 (0.7)	2.1 (0.7)	1.6 (0.7)	*p* < .001
Braden Friction subscale, *M* (*SD*)	1.9 (0.5)	1.9 (0.5)	1.6 (0.6)	*p* < .001

HAPrI, hospital-acquired pressure injury; arterial PaO2, arterial partial pressure of oxygen; MEWS, Modified Early Warning Scale; GCS, Glasglow Coma Scale; Riker, Riker Sedation and Agitation Scale; BMI, body mass index

### Predictive models

**Fig. 3:**
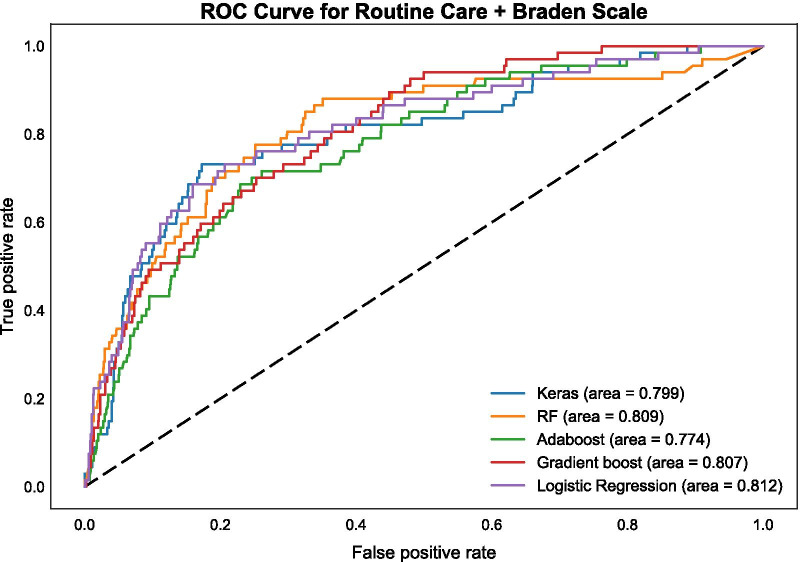
ROC curve for routine care + Braden Scale

**Fig. 4:**
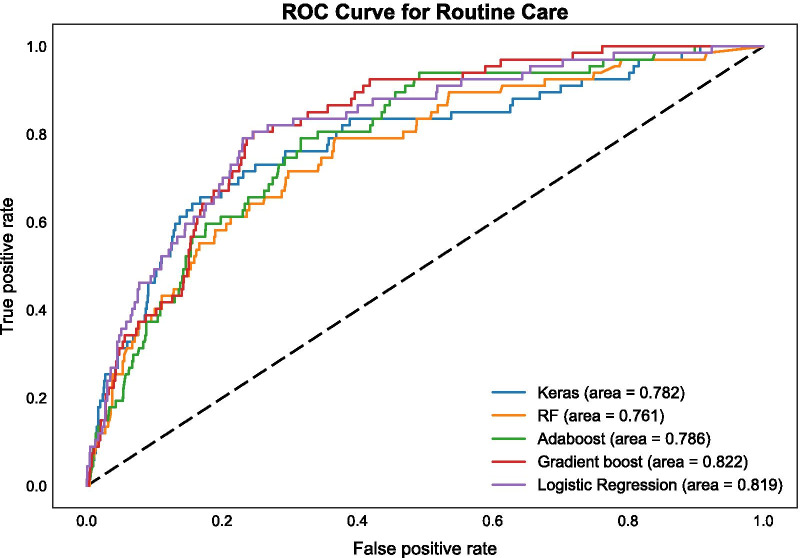
ROC curve for routine care

**Fig. 5:**
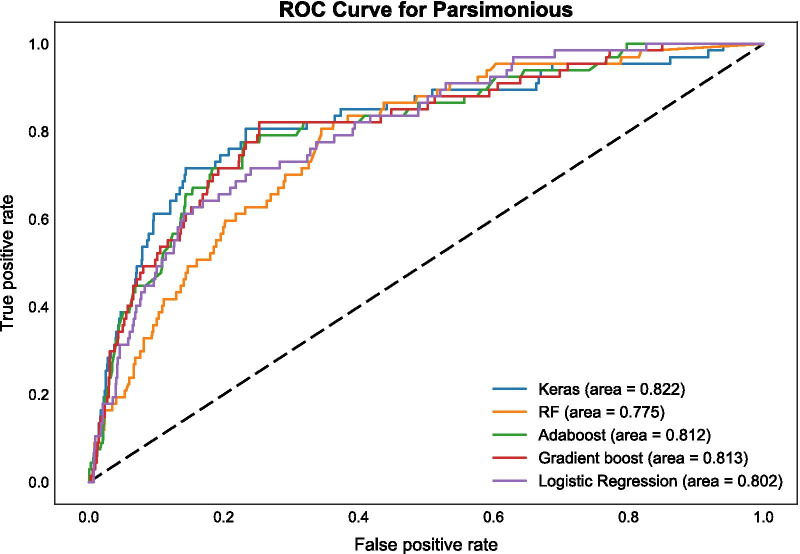
ROC curve for parsimonious

Confusion matrices for categorical (binary) prediction for each model and each set of features were produced and are available through the online supplement. Additionally, prediction probabilities vs. true event status are presented for each model as receiver operating characteristic (ROC) curves, with summary area under the curve (AUC) scores. The ROC curves using the Routine Care + Braden Scale data set for all five algorithms are presented in Fig. 3 (AUC range: 0.774–0.812); ROC curves using the Routine Care data set are shown in Fig. 4 (AUC range: 0.761–0.822); and ROC curves in the Parsimonious data set are presented in Fig. 5 (AUC range: 0.775–0.821). F_1_ scores for each type of algorithm across data sets (Routine Care + Braden Scale; Routine Care; Parsimonious) are shown in Table 3.

**Table 3:** F_1_ scores for algorithms

	Keras	RF	AdaBoost	Gradient boost	Logistic regression
Routine care + Braden Scale	0.28	0.36	0.29	0.28	0.31
Routine care	0.27	0.30	0.26	0.26	0.30
Parsimonious	0.34	0.26	0.33	0.35	0.28

RF, random forest

Although performance measures are generated on continuous scales, model performance is often evaluated within classes (from poor to excellent) and within the context of model deployment within the decision support setting. In this setting, model performance was comparable across the different data sets (Routine Care + Braden Scale; Routine Care; Parsimonious) and types of algorithms: neural networks (Keras), random forest, gradient boosting, adaptive boosting (AdaBoost), and multivariable logistic regression. Overall, the best performing model in the Routine Care + Braden Scale data set based on the AUC was logistic regression (AUC = 0.81), while random forest had the highest F_1_ score (F_1_ = 0.36). In the Routine Care data set, gradient boost generated the best discrimination based on the AUC (AUC = 0.82) and random forest and logistic regression tied for the highest F_1_ score (F_1_ = 0.30). Finally, Keras had the highest AUC at 0.82 in the parsimonious data set, while gradient boost had the highest F_1_ score at 0.35.

## Discussion

In this study, using data from surgical ICU patients within a single hospital system, we developed and compared models to predict HAPrIs in three data sets: Routine Care plus the Braden Scale, Routine Care, and a parsimonious set of five easily accessible predictor variables. This work represents a first step toward developing a model implementable in the EHR to provide real-time HAPrI risk assessment. In 2019, a guideline published by the NPIAP and its sister organizations, the European Pressure Injury Advisory Panel and the Pan Pacific Pressure Injury Alliance, recommended an automated approach to HAPrI risk assessment using data readily available in the EHR [5]. Advantages to harnessing data produced during routine care include more comprehensive evaluation of predictive factors related to HAPrI risk but missing from the Braden Scale (e.g., variables related to perfusion [3], severity of illness [26], and surgical factors [26–29]), the ability to update the risk prediction at frequent intervals with changes in patient status [9], and significantly decreasing the amount of time clinicians spend on data entry—currently, nurses manually record Braden Scale scores once per shift.

Our study results highlight the importance of considering class imbalance when evaluating model performance. The various types of models in our study all demonstrated good performance on the ROC curve. However, because HAPrIs are relatively rare, occurring in < 10% of ICU patients, continuous performance on the ROC curve may overestimate a model's utility due to the large number of true negatives [30]. F_1_ scores present an additional approach to evaluating the model's performance by utilizing a weighted average of precision and recall. Employing F_1_ scores was a strength in our study, enabling more accurate estimation of the model's true utility for predicting HAPrIs. For example, in the Routine Care + Braden Scale data set, gradient boosting was the best performer based on the area under the ROC curve, whereas logistic regression was the best performer based on F_1_ score. However, the gradient boosting algorithm was "accurate" because it primarily predicted the majority class—no HAPrI—and this is reflected in its lower F_1_ score.

Beyond classifying variables as missing or observed before imputation, it may be worthwhile considering modeling the observation in future work; for example, using a Heckman two-stage modeling approach (a model for observation and a model for outcome) [31]. However, we did not pursue this as we were not as interested in effect estimation (and therefore not as concerned with bias in estimated coefficients) but in building light and transportable models generating accurate prediction scores.

In our study, the inclusion of the Braden Scale modestly improved the models' performance. In contrast, a prior study found no improved performance when the Braden Scale was added to other variables obtained from routine-care documentation in the EHR [32]. Although including the Braden Scale did improve performance in our study, it requires clinicians to manually input six values during each shift. Reducing the time clinicians spend documenting is a worthy goal since documentation burden contributes to clinician burnout [33].

Models developed in the Parsimonious data set performed almost as well as the models developed using the larger number of predictor variables in the Routine Care data set. This finding is consistent with results from prior studies conducted among patients undergoing cardiovascular surgery [34, 35]. The Parsimonious model performance is encouraging in terms of clinical feasibility for eventual model implementation, demonstrating that a small group of informative variables can predict HAPrIs almost as well as a more comprehensive group of variables.

In our analysis, classic (parametric) multivariable logistic regression performed similarly to modern (nonparametric) machine learning models, including neural networks (Keras), random forest, gradient boosting, and adaptive boosting (AdaBoost). When performance is similar, logistic regression may be preferable to more complicated machine learning approaches for reasons of interpretability, computing power, and compactness, i.e., logistic regression is human-interpretable (coefficients give an immediate indication of size and direction of effect) and less computationally expensive. Furthermore, logistic regression models can be incorporated and run within most EHR systems (including EPIC^©^), simplifying model implementation. Our finding that multivariable logistic regression showed good performance in predicting HAPrI compared to other machine learning methods is consistent with a previous study [32].

Our study includes several limitations. Although we sought to include a comprehensive set of predictor variables, it is possible that other unexamined variables would improve predictive performance. For example, we did not consider some potential risk factors, including medications such as corticosteroids [36] or events like intra-operative blood loss [27], because those factors were more challenging to obtain in our EHR. Similarly, we chose not to collect data about preventive interventions like repositioning because prior studies have shown that documentation in the EHR does not necessarily reflect what was 'really' done [37]—particularly relevant in the setting of a busy ICU, where nurses have many competing priorities. Preventive interventions, especially repositioning, are also confounded by critical illness because repositioning is sometimes not possible due to hemodynamic instability.

## Conclusion

Overall, results from this study show the feasibility of using EHR data for accurately predicting HAPrIs and that good performance can be found with a small group of easily accessible predictor variables. Future study is needed to test the models in an external sample.

## Data Availability

The data used in this publication include protected health information, and therefore cannot be freely shared. Data sharing will be possible with case-by-case approval from our institution's Institutional Review Board; requests may be directed to the corresponding author.

## Abbreviations

CVICU: Cardiovascular surgical intensive care unit
EDW: Enterprise data warehouse
EHR: Electronic health record
HAPrI: Hospital-acquired pressure injury
ICU: Intensive care unit
ML: Machine learning
NPIAP: National Pressure Injury Advisory Panel
PrI: Pressure injury
ROC: Receiver operating characteristic
AUC: Area under the (ROC) curve
SICU: Surgical intensive care unit
SMOTE: Synthetic minority oversampling technique
